# The complete chloroplast genome sequence of *Kerria japonica* (L.) DC. ‘pleniflora’ (Rosaceae)

**DOI:** 10.1080/23802359.2019.1678433

**Published:** 2019-10-23

**Authors:** Yan Huo, Ming Yan, Xueqing Zhao, Zunling Zhu, Zhaohe Yuan

**Affiliations:** a College of Landscape Architecture, Co-Innovation Center for Sustainable Forestry in Southern China, Nanjing Forestry University, Nanjing, China;; b College of Forestry, Nanjing Forestry University, Nanjing, PR China;; c College of Arts and Design, Nanjing Forestry University, Nanjing, PR China

**Keywords:** *Kerria japonica*, *Kerria japonica* ‘pleniflora’, complete chloroplast genome, phylogenetic analysis

## Abstract

*Kerria japonica* (L.) DC., a monotypic species endemic to China and Japan, is an important ornamental shrub. In this study, we reported the complete chloroplast genome sequence of *K. japonica* (L.) DC. ‘pleniflora’ (GenBank accession number: MN418902) to provide genomic resources for its conservation. The total chloroplast (cp) genome is 160,007 bp in length, including a large single-copy region (LSC) of 87,672 bp and a small single-copy region (SSC) of 19,461 bp, which are separated by two inverted repeat (IR) regions of 26,437 bp. The overall guanine-cytosine (GC) content of the genome sequence is 34.0%. The cp genome encodes 132 unique genes, including 84 protein-coding genes, 40 tRNA genes, and 8 rRNA genes. Phylogenetic analysis of 25 cp genomes showed that *K. japonica* ‘pleniflora’ was most closely related to *K. japonica* and then had a close genetic relationship with *Neviusia cliftonii* in Rosaceae.

*Kerria japonica* (L.) DC. (Rosaceae) is a monotypic species endemic to China and Japon and distributed in certain mountainous areas (Chinese Academy of Science Flora of China Editorial Board 1985). It has high ornamental value for its bright yellow, rose-like spring flowers, and green winter stems. *K. japonica* ‘pleniflora’ is a popular double-flowered ornamental shrub that features rounded, pom-pom-like, yellow flowers, and it has gained the Royal Horticultural Society’s Award of Garden Merit (Zhang 2010). So far, although partial chloroplast DNA of *K. japonica* had been reported (Zhang et al. 2017), there have been no studies on the complete chloroplast genome of *K. japonica* or *K. japonica* ‘pleniflora’. Considering that the conservativity of cp DNA make it widely used in plant phylogeny (Olmstead and Palmer 1994.), we first report the complete chloroplast genome of *K. japonica* ‘pleniflora’, which will help the molecular and phylogenetical studies of this species.

Fresh leaves were collected from a single individual planted in Nanjing city (latitude: 32°07′86.1″N, longitude: 118°81′69.4″E), Jiangsu Province, China. The voucher specimen deposited in Nanjing Forestry University (accession number NFU19051101). The total genomic DNA was extracted using the modified CTAB method (Doyle 1987). Paired-end libraries were constructed and sequenced with an Illumina Hiseq 2500 platform (Nanjing, China) for paired-end 150 bp reads. Then, NOVOPlasty (Dierckxsens et al. 2017) was used for de novo assembly with *Rosa multiflora* (GenBank accession MG893867) as a reference. GeSeq (Tillich et al. 2017) was used for annotation and Geneious (Kearse et al. 2012) was used for inspecting the chloroplast genome.

The complete cp genome size of *K. japonica* ‘pleniflora’ is 160,007 bp in length, containing the large single-copy (87,672 bp), a small single-copy (19,461 bp), and two inverted repeats (26,437 pb) regions. The overall GC content of *K. japonica* ‘pleniflora’ cp genome is 34.0% and those in the LSC, SSC, and IR regions are 34.0%, 29.6%, and 42.6%, respectively. The cp genome encodes 132 unique genes, including 84 protein-coding genes (PCGs), 40 tRNA genes, and 8 rRNA genes. The tRNA genes are distributed throughout the whole genome with 24 in the LSC, 1 in the SSC, 15 in the IR regions, while rRNAs only situate in IR regions. One gene (*trnM-CAU*) has three copies. Twenty genes have two copies, which include eight PCGs, eight tRNA genes, and four all rRNA genes. Among the PCGs, three genes (*clpP*, *ycf3*, and *rps12*) contain two introns, and twelve different genes (*atpF, ndhA, ndhB, rpl2, rpoC1, rps16, trnA-UGC, trnG-UCC, trnI-GAU, trnK-UUU, trnL-UAA,* and *trnV-UAC*) have one intron each. The *rps12* is a trans-spliced gene with 5’ end located in the LSC region and the duplicated 3’ end in the IR regions.

Twenty-five cp genomes sequences were aligned with MAFFT (Katoh and Standley 2013), and the maximum likelihood (ML) tree was performed *via* IQ-TREE (Nguyen et al. 2015). The result revealed that *K. japonica* ‘pleniflora’ was most closely related to *K. japonica* and then had a close genetic relationship with *Neviusia cliftonii* in Rosaceae (Figure 1). The complete chloroplast sequence of *K. japonica ‘pleniflora’* will provide new insight into the evolution of genus *Kerria* and family *Rosaceae*.

**Figure 1.**
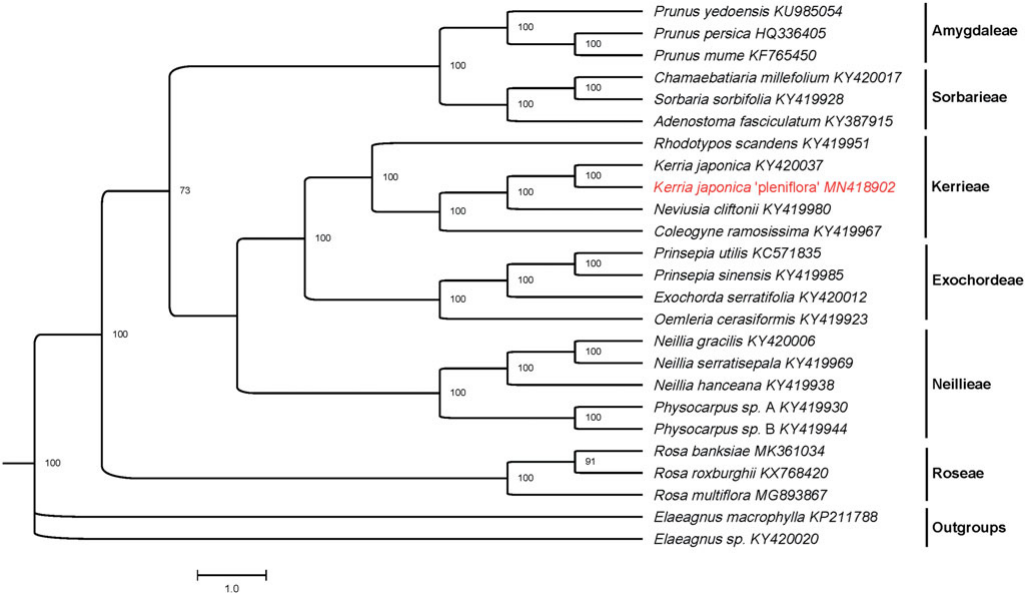
Maximum-likelihood (ML) tree based on the chloroplast genome sequences of 25 species. Numbers on the nodes are bootstrap values from 1000 replicates. *Elaeagnus macrophyll*a and *Elaeagnus sp.* were selected as outgroups.
